# AutoRadAI: a versatile artificial intelligence framework validated for detecting extracapsular extension in prostate cancer

**DOI:** 10.1093/biomethods/bpaf032

**Published:** 2025-04-26

**Authors:** Pegah Khosravi, Shady Saikali, Abolfazl Alipour, Saber Mohammadi, Maxwell Boger, Dalanda M Diallo, Christopher J Smith, Marcio C Moschovas, Iman Hajirasouliha, Andrew J Hung, Srirama S Venkataraman, Vipul Patel

**Affiliations:** Department of Biological Sciences, New York City College of Technology (City Tech), City University of New York (CUNY), Brooklyn, NY 11201, United States; PhD Programs in Biology and Computer Science, The Graduate Center, City University of New York (CUNY), New York, NY 10016, United States; BioMind AI Lab, New York City College of Technology (City Tech), City University of New York (CUNY), Brooklyn, NY 11201, United States; Global Robotics Institute, AdventHealth Celebration, Celebration, FL 34747, United States; BioMind AI Lab, New York City College of Technology (City Tech), City University of New York (CUNY), Brooklyn, NY 11201, United States; BioMind AI Lab, New York City College of Technology (City Tech), City University of New York (CUNY), Brooklyn, NY 11201, United States; Global Robotics Institute, AdventHealth Celebration, Celebration, FL 34747, United States; Department of Radiology, AdventHealth Celebration, Celebration, FL 34747, United States; Department of Radiology, AdventHealth Celebration, Celebration, FL 34747, United States; Global Robotics Institute, AdventHealth Celebration, Celebration, FL 34747, United States; Institute for Computational Biomedicine, Department of Physiology and Biophysics, Englander Institute for Precision Medicine, Meyer Cancer Center, Weill Cornell Medicine, New York, NY 10065, United States; Department of Urology, Cedars-Sinai Medical Center, Los Angeles, CA 90048, United States; Clinical Innovations, Promaxo Inc., Oakland, CA 94607, United States; Global Robotics Institute, AdventHealth Celebration, Celebration, FL 34747, United States

**Keywords:** artificial intelligence, deep learning, extracapsular extension, MRI, prostate cancer

## Abstract

Preoperative identification of extracapsular extension (ECE) in prostate cancer (PCa) is crucial for effective treatment planning, as ECE presence significantly increases the risk of positive surgical margins and early biochemical recurrence following radical prostatectomy. AutoRadAI, an innovative artificial intelligence (AI) framework, was developed to address this clinical challenge while demonstrating broader potential for diverse medical imaging applications. The framework integrates T2-weighted MRI data with histopathology annotations, leveraging a dual convolutional neural network (multi-CNN) architecture. AutoRadAI comprises two key components: ProSliceFinder, which isolates prostate-relevant MRI slices, and ExCapNet, which evaluates ECE likelihood at the patient level. The system was trained and validated on a dataset of 1001 patients (510 ECE-positive, 491 ECE-negative cases). ProSliceFinder achieved an area under the ROC curve (AUC) of 0.92 (95% confidence interval [CI]: 0.89–0.94) for slice classification, while ExCapNet demonstrated robust performance with an AUC of 0.88 (95% CI: 0.83–0.92) for patient-level ECE detection. Additionally, AutoRadAI’s modular design ensures scalability and adaptability for applications beyond ECE detection. Validated through a user-friendly web-based interface for seamless clinical integration, AutoRadAI highlights the potential of AI-driven solutions in precision oncology. This framework improves diagnostic accuracy and streamlines preoperative staging, offering transformative applications in PCa management and beyond.

## Introduction

The integration of artificial intelligence (AI) into radiology has transformed medical imaging by enabling faster, more accurate diagnoses and reducing the subjectivity inherent in human interpretation. AI applications range from detecting subtle abnormalities to predicting disease progression, offering unprecedented opportunities to enhance patient care and clinical decision-making [1]. Recent advances in machine learning (ML) and deep learning (DL) have driven this transformation, with convolutional neural networks (CNNs), a subset of DL algorithms, emerging as powerful tools for analyzing complex imaging data [2–4]. Despite this progress, fully automated, adaptive frameworks capable of addressing specific clinical challenges remain scarce. Existing AI algorithms often require manual preprocessing steps, specialized computational knowledge, or fail to integrate multimodal data such as imaging and histopathology. These limitations reduce their accessibility and practical utility in routine clinical workflows [5, 6]. To bridge this gap, there is a growing need for novel AI-driven algorithms that can seamlessly adapt to diverse clinical tasks, ensuring scalability, ease of use, and high diagnostic accuracy.

One critical application of AI in radiology is the preoperative staging of prostate cancer (PCa), particularly for detecting extraprostatic disease such as extracapsular extension (ECE). ECE, defined as cancer spreading beyond the prostate capsule, is a significant predictor of outcomes, affecting approximately one-third of newly diagnosed PCa cases. Its presence has been associated with an increased risk of positive surgical margins and early biochemical recurrence after radical prostatectomy [7–10]. Accurate preoperative ECE detection informs tailored treatment planning, balancing effective cancer removal with preservation of function [11].

The current standard for ECE detection relies on radiologists interpreting multiparametric magnetic resonance imaging (mpMRI) scans [12]. However, this approach is subjective and may lack consistency, even among experienced practitioners, due to the inherent complexity of prostate imaging. To overcome these limitations, AI-driven solutions have emerged as objective and scalable tools for ECE detection [13, 14]. While existing models show promise, they often rely solely on radiology imaging data, lack the ability to integrate histopathology, are validated on relatively small patient cohorts, and are not fully automated. These limitations reduce their comprehensiveness and robustness in clinical decision-making.

To address these challenges, we introduce AutoRadAI, an innovative AI-driven framework designed to provide fully automated analysis of PCa imaging, validated for the detection of ECE. AutoRadAI combines T2-weighted MRI data with histopathology annotations, leveraging a multi-convolutional neural network (multi-CNN) architecture. The framework comprises two core components: ProSliceFinder, which isolates relevant MRI slices, and ExCapNet, which assesses the likelihood of ECE presence. By leveraging this dual approach, AutoRadAI provides a detailed analysis that can assist clinicians in making informed surgical decisions [15, 16]. This modular architecture ensures adaptability, allowing AutoRadAI to scale for diverse clinical tasks beyond ECE detection.

In this study, AutoRadAI was validated using MRI scans from a large cohort of 1001 patients, achieving high diagnostic accuracy and demonstrating its ability to seamlessly integrate into clinical workflows through a user-friendly web-based interface. By automating complex imaging analyses and enabling data integration, AutoRadAI provides a scalable solution to enhance preoperative staging, improve precision oncology, and ultimately support better patient outcomes.

## Materials and methods

### Study population

Following institutional review board approvals, we retrospectively identified consecutive patients who underwent Robotic-Assisted Radical Prostatectomy between January 2018 and November 2023 and had a preoperative MRI of the prostate. Patients were classified into pT2 and pT3 groups based on the AJCC staging system. Eligibility criteria included undergoing surgical treatment, having a 3 T MRI of the prostate within one year prior to surgery (without an endorectal coil), and having a pathological diagnosis of PCa on surgical specimens. Exclusion criteria were the absence of preoperative MRI, MRI images older than one year, and prior treatment for PCa, though treatments for benign prostatic hyperplasia or bladder outflow obstruction were allowed. MRIs from multiple centers, with various machines, were collected and uploaded to our PACS for storage and data extraction. Surgeries were performed by a robotic surgery expert, and a genitourinary pathologist with over a decade of experience evaluated the surgical pathology.

### Data preparation

**Figure 1.**
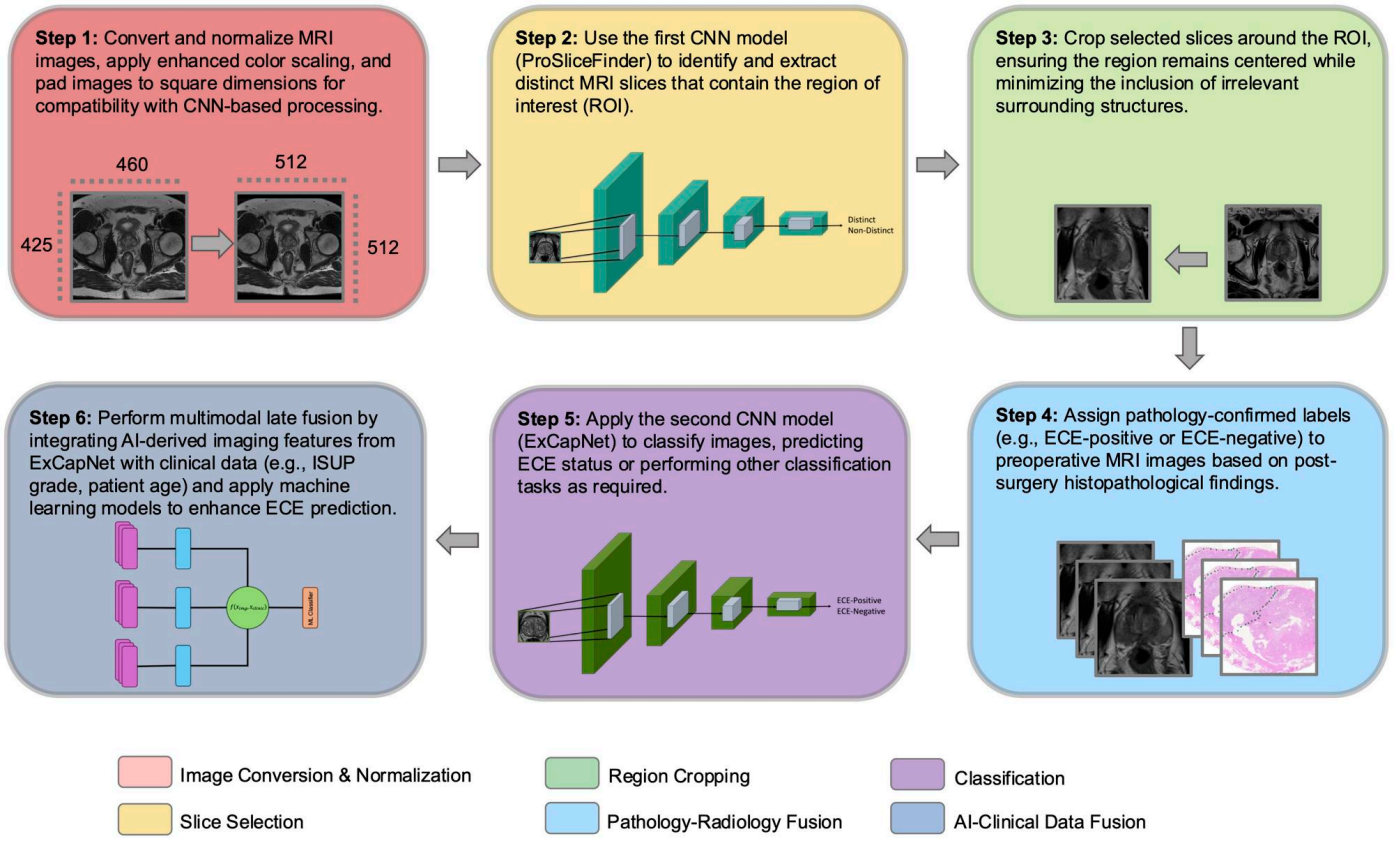
Schematic representation of the AutoRadAI pipeline. The workflow begins with MRI preprocessing, where images undergo intensity normalization, enhanced color scaling, and padding to ensure compatibility with CNN-based processing. ProSliceFinder then selects MRI slices that contain the prostate region, filtering out irrelevant anatomy. Cropping is applied to center the prostate while minimizing surrounding structures. Pathology-confirmed labels from post-surgery histopathological findings are assigned to preoperative MRI scans. ExCapNet classifies these labeled slices to predict ECE status or perform other classification tasks. Finally, multimodal late fusion integrates AI-derived imaging features with clinical data, such as ISUP grade and patient age, using machine learning models to enhance ECE prediction. The diagram illustrates the sequential steps, with outputs from preprocessing feeding into ProSliceFinder, followed by ExCapNet and the final AI-clinical data fusion.

We curated a comprehensive dataset of preoperative MRI scans from 1001 PCa patients, annotated by fellowship-trained urologists. This dataset includes T2-weighted (T2W) MR images labeled with pathology results (Fig. 1) indicating the presence (ECE positive, 510 patients) or absence (ECE negative, 491 patients) of ECE. To ensure uniformity, we standardized the images to maintain consistent dimensions. Additionally, we developed scripts for converting and padding the images to a resolution of 512 × 512 pixels, facilitating compatibility across analyses (Fig. 1). We then adopted a multislice approach, processing multiple MRI slices per patient to increase data volume and granularity (Fig. 1). This approach provides a comprehensive anatomical representation, supporting more accurate diagnosis and analysis [17]. From the total images in our dataset, we created two sub-datasets to train distinct models: ProSliceFinder and ExCapNet. The ProSliceFinder sub-dataset contains 3660 images, labeled by expert radiologists as 1828 “Distinct slices” displaying the prostate gland and 1832 “Non-Distinct slices” depicting surrounding pelvic anatomy (Table 1). Distinct slices were defined based on anatomical visibility of the prostate gland, as determined by expert radiologists. No additional image quality thresholds (e.g. motion artifacts, signal-to-noise ratio) were applied during selection, though the scans included met standard clinical imaging protocols.

**Table 1.** Summary of datasets for training, validation, and testing of ProSliceFinder (distinct/non-distinct slices) and ExCapNet (ECE-negative/ECE-positive images).

Model	Dataset	Number of Images	Details
ProSliceFinder (3660 Images)	Training	2736	1374 distinct/1362 non-distinct
	Validation	457	224 distinct/233 non-distinct
	Test	467	230 distinct/237 non-distinct
ExCapNet (6162 Images)	Training	4200	2171 ECE-negative/2029 ECE-positive from 701 patients (360 ECE-positive/341 ECE-negative)
	Validation	1340	681 ECE-negative/659 ECE-positive from 200 patients
	Test	622	329 ECE-negative/293 ECE-positive from 100 patients

The ExCapNet sub-dataset consists of 6162 MRI images, with 3181 labeled as ECE-negative and 2981 as ECE-positive based on post-surgery pathology reports (Table 1).

Before classification by ExCapNet, images were preprocessed to focus on the relevant anatomical region. A script cropped images around the prostate gland, ensuring the algorithm analyzes the tumor's presence within or adjacent to the prostate gland or seminal vesicle, excluding other areas (Fig. 1). This preprocessing prepares the images for final classification into ECE-positive and ECE-negative categories (Fig. 1).

### Multi-CNN model characteristics

Our DL approach is based on a novel, lightweight multi-convolutional neural network (multi-CNN) architecture designed to detect distinct MRI slices and identify ECE in PCa patients. This multi-CNN model integrates two sequential CNN algorithms: ProSliceFinder, which identifies the most informative MRI slices where the prostate gland is clearly visible (Distinct slices), and ExCapNet, which classifies patients into ECE-positive or ECE-negative groups based on these slices. Both CNNs were trained from scratch, foregoing pre-trained models to allow a deeper, dataset-specific understanding and reduce the risk of overfitting.

#### CNN architecture and key layers

Our CNN architecture balances high predictive accuracy with minimal overfitting risk by leveraging depthwise separable convolutions, which decompose the standard convolution operation into separate depthwise and pointwise convolutions. This process reduces computational complexity from $O(k^{2}\cdot c\cdot d\cdot h\cdot w)$ in standard convolutions to $O(k^{2}\cdot c\cdot h\cdot w+c\cdot d\cdot h\cdot w)$, allowing the model to operate more efficiently. In practical terms, this means that AutoRadAI can process high-resolution imaging data with greater speed and less computational demand, an important factor for clinical deployment.

Each convolutional layer is followed by batch normalization and a ReLU activation function. Batch normalization standardizes the input x across each batch as $\hat{x}=\frac{x-\mu }{\sqrt{\sigma^{2}+\epsilon }}\;$where μ and $\sigma^{2}$ represent the mean and variance of the batch, and ϵ is a small constant added for numerical stability. This normalization accelerates convergence and acts as a regularizer, reducing internal covariate shift. The ReLU activation function, ReLU(x)=max(0,x), introduces non-linearity, enabling the network to learn complex, hierarchical features. This allows the model to detect complex patterns in imaging data that might be missed by traditional algorithms.

#### Pooling and fully connected layers with dropout

Each convolution block is accompanied by a max pooling layer with stride 2, reducing the spatial dimensions (from h×w to $\frac{h}{2}\;$× $\frac{w}{2}$) and thereby retaining key features while enforcing spatial invariance. This step reduces the risk of overfitting by ensuring that the network focuses on prominent features. Each max pooling layer reduces the image size by half, retaining only the most important features. This simplifies the data while preserving clinically relevant patterns.

The fully connected layers at the network’s end consist of two dense layers, with a dropout layer (60% dropout probability) in between to prevent overfitting. The dropout layer functions by randomly zeroing out a portion of the activations, calculated as y=f(W⋅(x⊙mask)) where ⊙ denotes element-wise multiplication with a binary mask sampled from a Bernoulli distribution, and W is the weights matrix. Dropout layers reduce overfitting by preventing the model from becoming too dependent on any one feature. This leads to more reliable generalization when analyzing unseen patient data.

#### Loss function and optimization strategy

For classification, we employ the cross-entropy loss $L=-\sum_{i=1}^{C}t_{i}\;\cdot \;\log(p_{i})$ where $p_{i}$​ is the predicted probability of class i and C is the number of classes and $t_{i}$ represents the true label.

Optimization is conducted with the RMSprop algorithm, which adapts the learning rate based on the moving average of squared gradients $\theta_{t+1}=\theta_{t}-\frac{\eta }{\sqrt{E[g^{2}{]}_{t}+\epsilon }}\;\cdot {\;g}_{t}$ where $E[g^{2}{]}_{t}$ is the running average of squared gradients, η is the learning rate, and ${\;g}_{t}$​ is the gradient. This technique automatically adjusts how quickly the model learns, helping avoid overcorrection or instability during training. Additionally, weight decay regularization is applied, penalizing large weights to reduce overfitting, expressed as $L_{\mathrm{total}}=L+\lambda \sum_{i}||w_{i}|{|}^{2}$ where λ is the regularization coefficient, and $||w_{i}|{|}^{2}$ is the squared norm of the weights. Weight decay regularization penalizes overly complex models to prevent overfitting. This improves the model’s ability to generalize its predictions to new patient scans, which is essential for clinical reliability.

#### Implementation details and data processing

**Figure 2.**
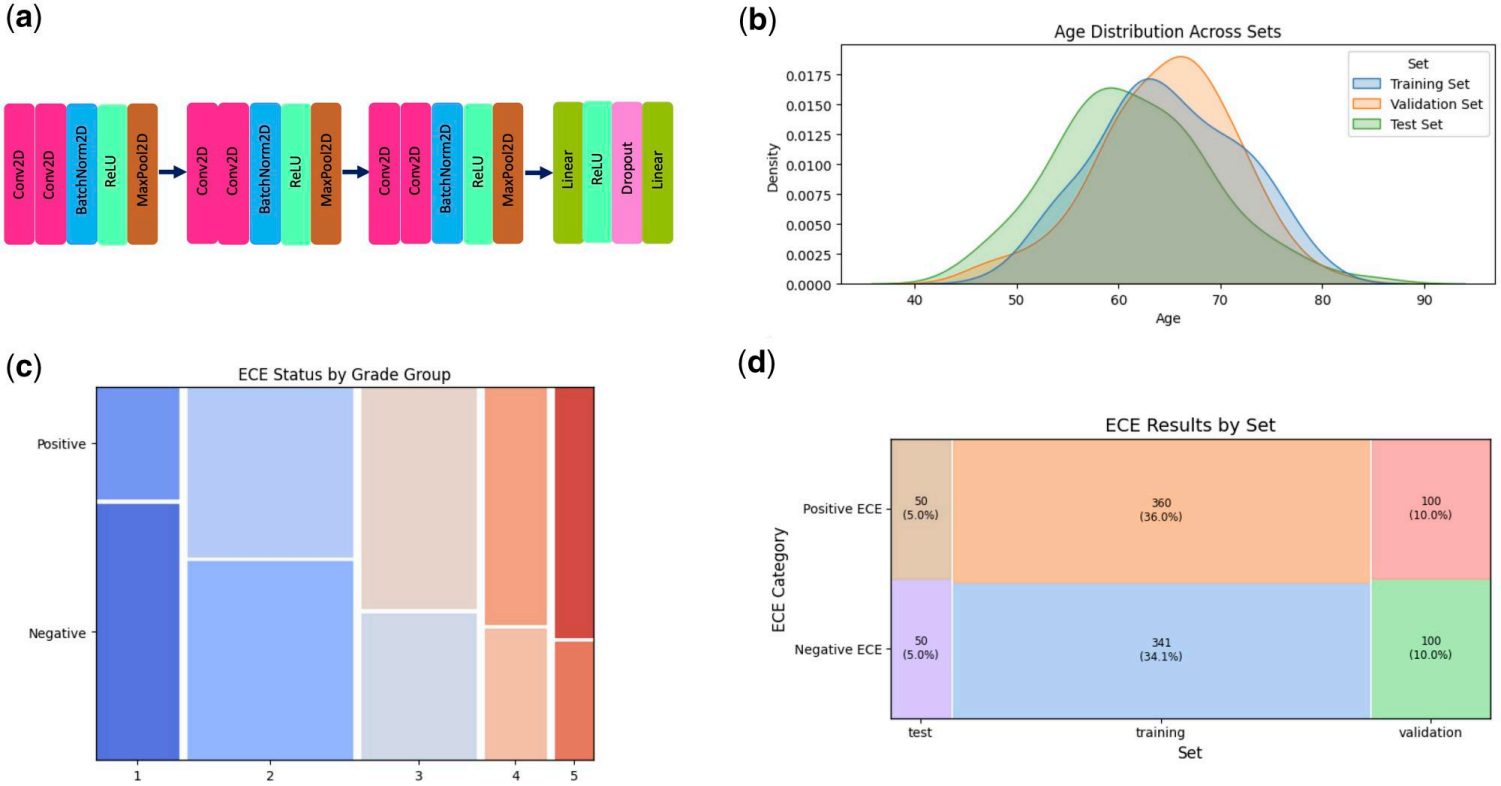
Architectural diagram of the CNN model utilizing depthwise Conv2D layers (**a**). Age distribution of patients across the training, validation, and test sets (**b**). Distribution of ECE status (positive/negative) across the five pathology grades (**c**). Distribution of the number and percentage of patients across the training, validation, and test sets based on a 70:20:10 split (**d**).

The network includes three primary convolution blocks, each followed by batch normalization, ReLU activation, and max pooling. Channels increase from 3 to 12, then to 24, and finally to 36, with spatial resolution halved at each pooling layer. The dense segment includes two fully connected layers separated by a dropout layer set at 60%, with the final layer producing a probability distribution across two classes. Image inputs are resized to 512 × 512 pixels, randomly flipped, and normalized. Data are loaded with PyTorch's DataLoader, utilizing shuffled batches of 64 for training and 32 for validation. Optimization employs RMSprop with an initial learning rate of 0.00001, an alpha of 0.9, and a momentum of 0.5, while weight decay mitigates overfitting (Fig. 2a).

### Data distribution

As shown in Fig. 2b, our dataset is balanced across training, validation, and testing sets, accounting for key variables such as patient demographics (e.g. age) and pathological grades related to ECE outcomes (Fig. 2c). The dataset includes 4200 images from 701 patients for training, 1340 images from 200 patients for validation, and 622 images from 100 patients for testing (Fig. 2d). The proportions of each subset were calculated as $\mathrm{Training}\;\mathrm{Proportion}=\frac{N_{\mathrm{train}}}{N_{\mathrm{total}}}$, $\mathrm{Validation}\;\mathrm{Proportion}=\frac{N_{\mathrm{val}}}{N_{\mathrm{total}}}$, $\mathrm{Testing}\;\mathrm{Proportion}=\frac{N_{\mathrm{test}}}{N_{\mathrm{total}}}$ where $N_{\mathrm{train}}=4200,\;N_{\mathrm{val}}=1340,\;N_{\mathrm{test}}=622$, and $N_{\mathrm{total}}=6162$. This stratified distribution ensures demographic and pathological grade homogeneity, enhancing fairness and representativeness across subsets. Such an approach provides a robust foundation for model training and evaluation by accurately reflecting the larger dataset.

### Statistical tests and predictive models

We investigated the relationship between patient age and preoperative ISUP grade group using a combination of ML and DL approaches. Clinical features were preprocessed according to model requirements. Patient age was used in its original numeric form without normalization, as tree-based models (Decision Tree, Random Forest, XGBoost) are not sensitive to feature scaling. The Preoperative ISUP grade group was transformed using one-hot encoding (with the first category dropped to prevent multicollinearity). This encoding allowed for efficient integration with AI-derived predictions during model training. ML models included Decision Tree Classifier (DTC), Random Forest, and XGBoost, which were implemented in Scikit-learn (Python 3.10.12), while DL models were developed using the PyTorch framework (version 1.9.0). To train and validate the models, the dataset was split into a 70/30 training/testing configuration. Model performance was assessed using key statistical metrics, including Specificity, Sensitivity, Accuracy (ACC), and ROC curve analysis to calculate the AUC (Area Under the Curve).

In the evaluation of model performance, Sensitivity (True Positive Rate) is calculated as $\mathrm{Sensitivity}\;=\frac{\mathrm{True}\;\mathrm{Positives}\;(TP)}{\mathrm{True}\;\mathrm{Positives}\;(TP)\;+\;\mathrm{False}\;\mathrm{Negatives}\;(FN)}$, Specificity (True Negative Rate) is defined as $\mathrm{Specificity}\;=\frac{\mathrm{True}\;\mathrm{Negatives}\;(TN)}{\mathrm{True}\;\mathrm{Negative}\;(TN)\;+\;\mathrm{False}\;\mathrm{Positives}\;(FP)}$, and The Accuracy (ACC) metric is represented by $\mathrm{Accuracy}\;=\frac{TP\;+\;TN}{TP\;+\;TN\;+\;FP\;+\;FN}$. The ROC curve, which plots the True Positive Rate against the False Positive Rate, provides a visual measure of model performance. The AUC represents the discriminatory ability of the model, with values closer to 1 indicating superior performance.

To further validate the model, we compared its predictions against radiologists with various levels of expertise in prostate MRI interpretation. For this comparison, we utilized the Jaccard Index and Cohen’s Kappa coefficient to measure agreement. The Jaccard Index is calculated as $\mathrm{Jaccard}\;\mathrm{Index}=\frac{|A\cap B|}{|A\cup B|}$ where A and B represent the prediction sets of the model and radiologist annotations, respectively. Cohen’s Kappa, which adjusts for chance agreement, is defined by $\kappa =\frac{p_{o}-p_{e}}{1-p_{e}}$ where $p_{o}$​ is the observed agreement between the model predictions and radiologist annotations and $p_{e}$ represents the expected agreement by chance.

This analytical framework and detailed statistical measures ensure a rigorous evaluation of model performance, establishing its reliability in comparison to radiologist benchmarks and its potential for practical application in clinical settings.

### Data and code availability

This study was approved by the AdventHealth IRB (protocol number 3009855250, approved on 06/15/2023, and protocol number 2039092-4, approved on 02/25/2025). A Data Use Agreement between CUNY and AdventHealth enabled the use of medical imaging data for research at CUNY. The source code for AutoRadAI is also accessible via GitHub https://github.com/PKhosravi-CityTech/AutoRadAI.

## Results

### Distinct scans and ECE detection evaluation

Our analysis of the AutoRadAI system—a comprehensive pipeline that integrates a multi-CNN architecture along with additional scripts to automate the process from raw patient data to final classification results—highlights its effectiveness in detecting ECE in preoperative MRI scans of PCa patients.

**Figure 3.**
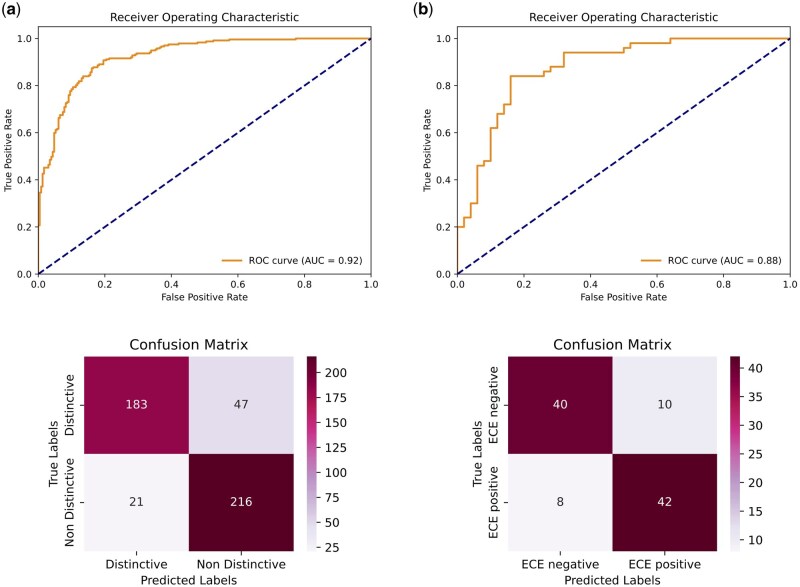
Performance of ProSliceFinder (**a**) and ExCapNet (**b**) for detecting Distinct (visible prostate gland) slices and ECE detection, respectively using ROC curves and confusion matrix.

The ProSliceFinder algorithm, designed to analyze individual image slices, demonstrated strong performance on a blind test set of 467 MRI slices, achieving an AUC of 0.92 (95% CI: 0.89–0.94), Sensitivity of 0.91, Specificity of 0.80, and ACC of 0.85. Figure 3a's ROC curve and confusion matrix illustrate high diagnostic accuracy, with 183 distinct and 216 non-distinct slices correctly identified. These performance metrics were derived from the repeated cross-validation approach, ensuring the robustness of the results.

The ExCapNet model, which assesses data at the patient level, achieved an AUC of 0.88 (95% CI: 0.83–0.92), Sensitivity of 0.84, Specificity of 0.80, and ACC of 0.82 on a blind test set of 100 randomly selected patients (Fig. 3b). The ROC curve and confusion matrix reveal effective ECE classification, with 42 true positives and 40 true negatives. This model leverages slice-level insights from ProSliceFinder to inform patient-level ECE predictions.

The performance of the ProSliceFinder and ExCapNet models was evaluated using Repeated Cross-Validation and Bootstrap Aggregation (Bagging). These techniques involve training the models multiple times on different subsets of the data and averaging the results to ensure robust performance estimates and minimize variance. The Repeated Cross-Validation approach was used to mitigate the impact of random variations in training, while Bootstrap Aggregation helped to stabilize model performance by aggregating predictions from multiple trained models. This rigorous process, which includes repeated training and testing on a blind test set, ensures that the reported performance metrics are not overly dependent on a single random initialization or split of the data, thereby providing a more reliable and unbiased evaluation of model effectiveness.

### Predicting ECE using conventional ML algorithms

**Figure 4.**
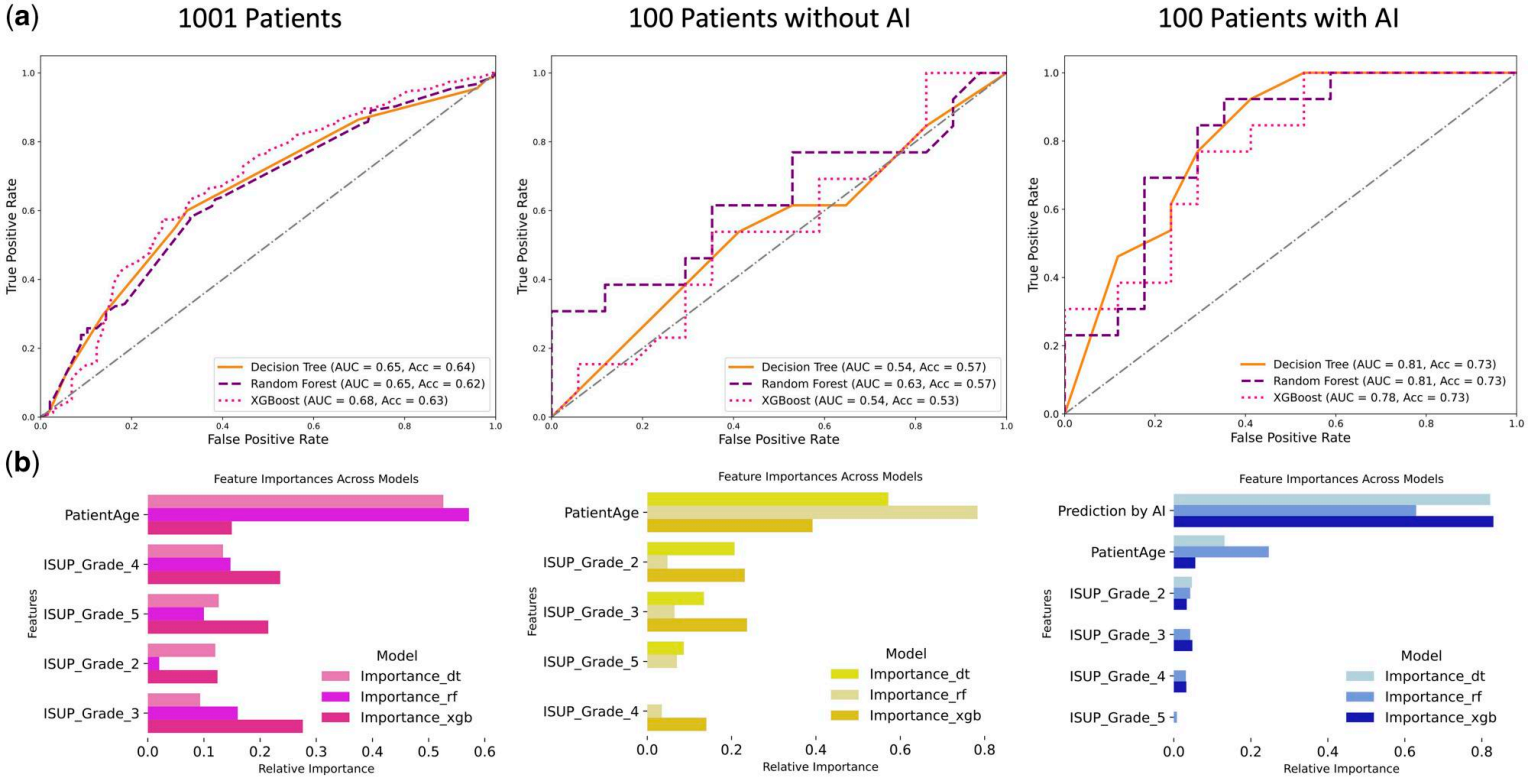
Analytical performance of Decision Tree, Random Forest, and XGBoost models using histopathological grades and patient age for predictive analysis. Comparative evaluation of predictive accuracy across the full dataset of 1001 patients and a subset of 100 patients, highlighting the impact of incorporating AI-derived features (**a**). Key features identified by each model as critical for predictive performance, presented by model and dataset (**b**).

We conducted a comparative analysis of three ML models—Decision Tree, Random Forest, and XGBoost—to assess their efficacy in predicting ECE using ISUP grade group and patient age across a cohort of 1001 patients. In this process, we utilized RandomizedSearchCV to optimize hyperparameters, combined with cross-validation, which ensures robust performance evaluation by reducing overfitting and accounting for variability in the training process. Additionally, for the Random Forest model, we leveraged Bagging, a method that trains multiple decision trees on bootstrapped subsets of the data and aggregates their predictions to improve generalization. For the 1001-patient cohort, the Decision Tree achieved an ACC of 0.64 and an AUC of 0.65, Random Forest an ACC of 0.62 and AUC of 0.65, and XGBoost led with an ACC of 0.63 and AUC of 0.68 (Fig. 4a). To further evaluate model performance, we assessed the impact of integrating AI-derived features with additional clinical information in the blind test set of 100 randomly selected patients (Fig. 4). In the 100-patient cohort without AI-derived features (Fig. 4a), performance metrics declined across all models, highlighting the challenge of predicting ECE with limited data. However, incorporating AI-derived features significantly improved model performance, with Decision Tree, Random Forest, and XGBoost AUC values rising to 0.81, 0.81, and 0.78, respectively, and all achieving an ACC of 0.73 (Fig. 4a).

This improvement underscores the additive value of AI-driven insights in enhancing model predictions. Figure 4b presents feature importance rankings, which clearly show that AI-derived predictions emerged as the most significant determinant of ECE, surpassing traditional clinical markers such as patient age and ISUP grade. This demonstrates the capability of AI-derived features to enhance model performance, especially when conventional data is limited. Integrating AI-derived features with traditional clinical data significantly improves predictive accuracy, compensating for lower accuracy typically observed in large, heterogeneous datasets.

This approach aligns with current trends in AI research, emphasizing the integration of multimodal data to enhance diagnostic precision and optimize patient care strategies. By enriching the predictive landscape with AI insights, our models not only improve accuracy but also advance the broader goal of personalized medicine, facilitating more informed and precise patient care strategies [18].

### Comparative performance: radiologist assessment vs. AutoRadAI

**Figure 5.**
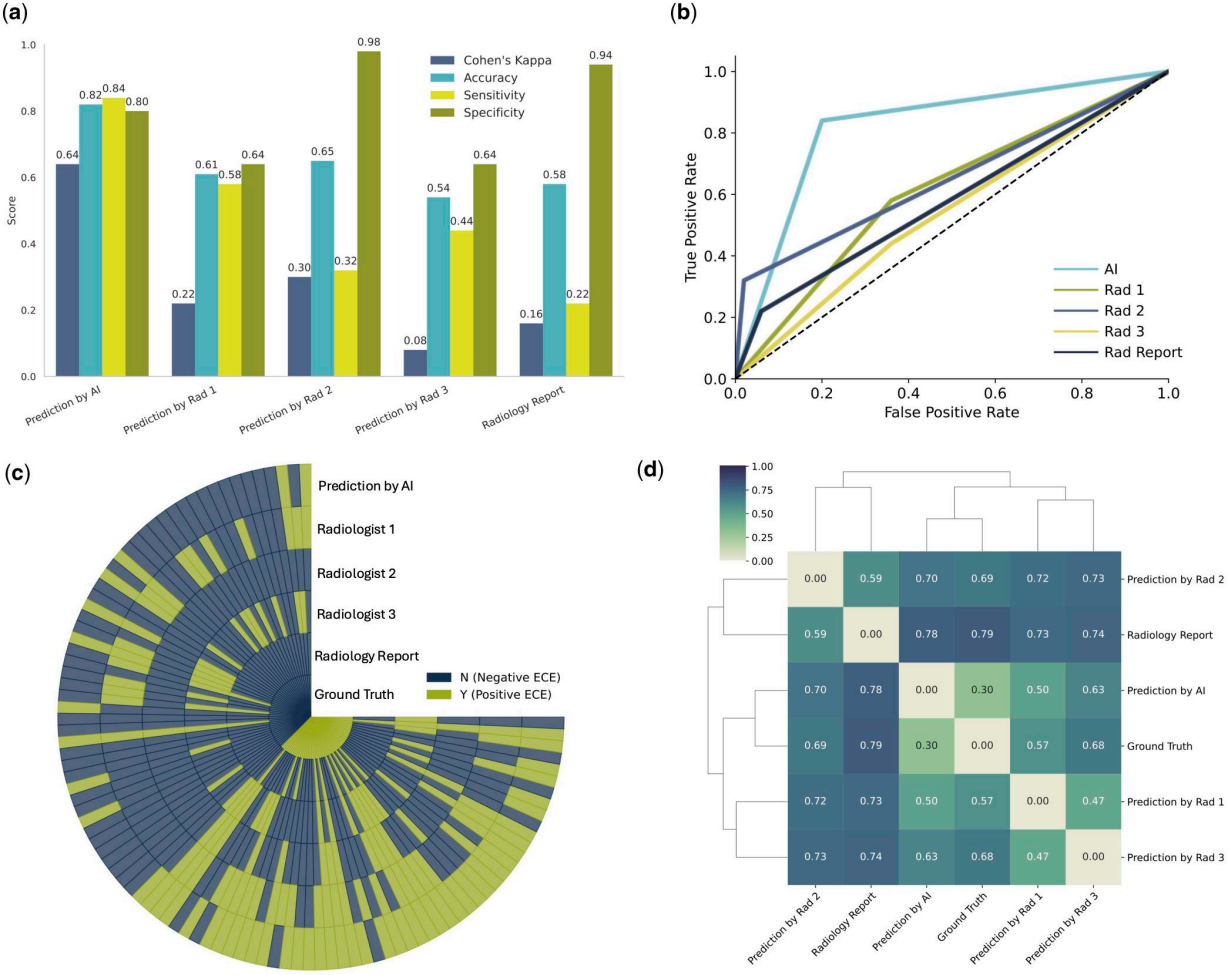
Performance comparison of AutoRadAI for ECE detection in MRI scans against radiologists, including Cohen’s Kappa, Sensitivity, Specificity, and Accuracy metrics (**a**), AUC (**b**), Circular heatmap (**c**), and Dendrogram (**d**) for AI versus radiologist predictions.

In the final phase of our study, three board-certified radiologists independently assessed ECE presence in MRI scans of 100 patients, without knowledge of pathology outcomes or existing radiology reports, to compare their diagnostic accuracy with AutoRadAI against the pathology reports (Fig. 5).

Radiologists were not given additional training or standardized criteria specific to this study. Instead, they were instructed to rely on their own clinical experience and judgment to identify ECE based solely on the MRI images, without access to prior reports or pathology outcomes. This approach reflects real-world variability in radiologist interpretation and allows for a more authentic comparison to the AI system’s performance.

The radiologists' diagnostic performance recorded ACCs of 0.61, 0.65, and 0.54, with the formal radiology report yielding an ACC of 0.58 (Fig. 5a and b). These results indicate that ECE determination through imaging remains challenging even for seasoned practitioners. In contrast, AutoRadAI demonstrated superior classification capabilities with an ACC of 0.82. Its agreement with pathology results, measured by Cohen’s kappa score, was substantial (kappa = 0.64), significantly higher than the individual radiologists (kappa = 0.22, 0.30, 0.08) and the radiology report (kappa = 0.16), indicating improved reliability and accuracy (Fig. 5a).

Figure 5c presents a circular heatmap and Fig. 5d shows a dendrogram. The heatmap illustrates concordance and discordance among assessments by the radiologists, the formal radiology report, and the confirmed pathology cases, highlighting diagnostic alignment.

The dendrogram, constructed using the Jaccard distance index, clusters the radiologists based on the similarity of their diagnostic decisions and contrasts these with AutoRadAI’s outcomes. Jaccard distances of 0.57, 0.69, 0.68, and 0.79 were observed among the radiologists, with the AI system showing a closer alignment to the ground truth (Jaccard distance = 0.30), revealing distinct diagnostic approaches and accuracy levels (Fig. 5d). These analyses, particularly the stark contrast in performance between the human experts and AutoRadAI, underline AI's potential to enhance medical diagnostics by providing more reliable and accurate ECE assessments.

## Discussion

Accurate preoperative prediction of ECE is essential for optimizing surgical planning and improving oncological outcomes in PCa. While clinical nomograms for ECE prediction exist [19–22], they were developed before the widespread adoption of mpMRI, highlighting a gap in leveraging advanced imaging techniques for risk stratification. MRI has demonstrated high sensitivity (>90%) in detecting clinically significant PCa [23], making it a valuable modality for preoperative assessment. Recent advances in artificial intelligence (AI) have further enhanced ECE prediction, with ML and DL models outperforming radiologists in specific tasks. For instance, an AI model trained on MRI scans of 849 patients achieved a moderate AUC (95% CI, 0.63–0.86) [15], while a logistic regression model using 139 patients demonstrated an AUC of 0.93 and an accuracy of 0.78 [24]. Additionally, an SVM model trained on 193 patients achieved an accuracy of 0.79 and an AUC of 0.80 on an independent test dataset [25].

Our study builds upon these findings by introducing AutoRadAI, an AI-driven framework that integrates multi-slice image analysis for ECE detection. The ProSliceFinder model enhances the selection of informative MRI slices, optimizing the quality of data input for ECE classification. At the patient level, ExCapNet achieved an AUC of 0.88 and an accuracy of 0.82, surpassing the diagnostic performance of radiologists and traditional radiology reports. The ability of AutoRadAI to standardize image interpretation while leveraging multi-slice analysis suggests that AI-driven approaches could improve the consistency of preoperative staging. Additionally, integrating texture analysis-based radiomics could further refine the detection of subtle extracapsular invasion, reinforcing the potential of multimodal AI frameworks in PCa diagnosis [26]. Our ML models incorporated clinical variables such as age and Gleason score, underscoring the synergy between AI-derived features and conventional clinical risk factors in predicting ECE [27].

Despite these promising results, this study has several limitations. One major limitation is the absence of spatial localization in AutoRadAI’s predictions. Although the model identifies the presence of ECE, it does not provide visual cues regarding its precise location on MRI scans, which is a crucial factor for surgical decision-making. Explainable AI (XAI) methods, such as Grad-CAM or saliency maps, could enhance the interpretability of AutoRadAI by highlighting regions of interest in the MRI slices that contributed to the prediction. Future work will focus on integrating XAI-based visualization techniques to improve model transparency and clinical usability.

Another important consideration is external validation. While this study was conducted using data from AdventHealth, a multi-center healthcare system with diverse imaging protocols and patient populations, further validation using independent datasets from additional institutions will be valuable to confirm the robustness of AutoRadAI and ensure its broad clinical applicability. Additionally, while the ML models in this study relied on age and ISUP grade as predictive features, other clinically relevant parameters, such as prostate-specific antigen levels, PIRADS scores, and lesion size, may further improve ECE prediction. However, these variables were not included due to limited access to comprehensive clinical data. Future iterations of AutoRadAI will incorporate these additional features to enhance its predictive power. A further limitation relates to MRI sequence selection which is exclusively on T2-weighted axial MRI images. Incorporating additional mpMRI sequences, such as diffusion-weighted imaging and apparent diffusion coefficient maps, could improve model accuracy and provide a more comprehensive representation of extracapsular disease. Expanding the training dataset to include multiple MRI modalities will be an important direction for future research. Finally, while AutoRadAI is accessible via a web-based interface, its usability and integration into routine clinical workflows remain to be evaluated. A critical next step will involve usability studies with radiologists and urologists to assess how effectively the AI system can be incorporated into decision-making processes. We are actively collaborating with hospitals to facilitate these evaluations and gather real-world clinical insights. Future developments will include enhanced visualization tools, interactive AI-assisted decision support features, and real-time clinical feedback mechanisms to optimize AutoRadAI for practical application.

In conclusion, this study demonstrates the feasibility and clinical potential of an AI-driven framework for ECE detection, leveraging a multi-slice analysis approach to enhance diagnostic accuracy. AutoRadAI outperformed radiologists, underscoring its value as a standardized, scalable tool for improving preoperative PCa assessment. Beyond its diagnostic capabilities, this framework represents a step toward integrating AI into precision oncology, offering a reproducible and accessible solution for real-world clinical use. While challenges such as spatial localization, external validation, and additional imaging sequences remain, these provide a roadmap for future advancements. By incorporating explainable AI, expanding validation efforts, and integrating additional clinical and imaging features, AutoRadAI is poised to become a transformative tool in PCa management, ultimately improving patient outcomes through more accurate and personalized treatment planning.
